# A case of severe psittacosis in a hemodialysis patient—the critical role of detailed medical history and next-generation sequencing

**DOI:** 10.3389/fmed.2026.1825118

**Published:** 2026-05-22

**Authors:** Yuanzi Liang, Junqi Hu, Zhiqun Wang

**Affiliations:** 1 Department of Radiology, Aerospace Center Hospital, Beijing, China; 2 Department of Nephrology, Aerospace Center Hospital, Beijing, China

**Keywords:** hemodialysis, metagenomic next-generation sequencing (mNGS), neuropsychiatric manifestations, parrot exposure, psittacosis

## Abstract

An 80-year-old male patient on maintenance hemodialysis was admitted with “high fever and cough.” Pulmonary imaging suggested pneumonia, but his condition deteriorated rapidly despite empirical broad-spectrum antimicrobial therapy (covering bacteria, atypical pathogens, and fungi), progressing to respiratory failure and delirium. He was transferred to the intensive care unit for continuous renal replacement therapy. Routine microbiological tests (blood culture, sputum culture, respiratory pathogen PCR) were all negative. Detailed history revealed that the patient had kept a parrot for over a month prior to illness onset. Metagenomic next-generation sequencing of blood and sputum specimens detected abundant *Chlamydia psittaci* sequences. Following confirmation, treatment was adjusted to oral minocycline combined with intravenous azithromycin. The patient’s temperature gradually normalized, neuropsychiatric symptoms resolved, and pulmonary imaging showed marked improvement, ultimately leading to successful discharge. This case highlights the importance of considering zoonotic pathogens in immunocompromised patients with refractory pneumonia. Detailed history-taking and metagenomic next-generation sequencing (mNGS) technology are crucial for early diagnosis. Early use of mNGS should be strongly considered in immunocompromised patients with severe pneumonia unresponsive to empiric therapy and negative routine workup, particularly when epidemiological clues such as bird exposure are present.

## Case report

**Figure 1:**
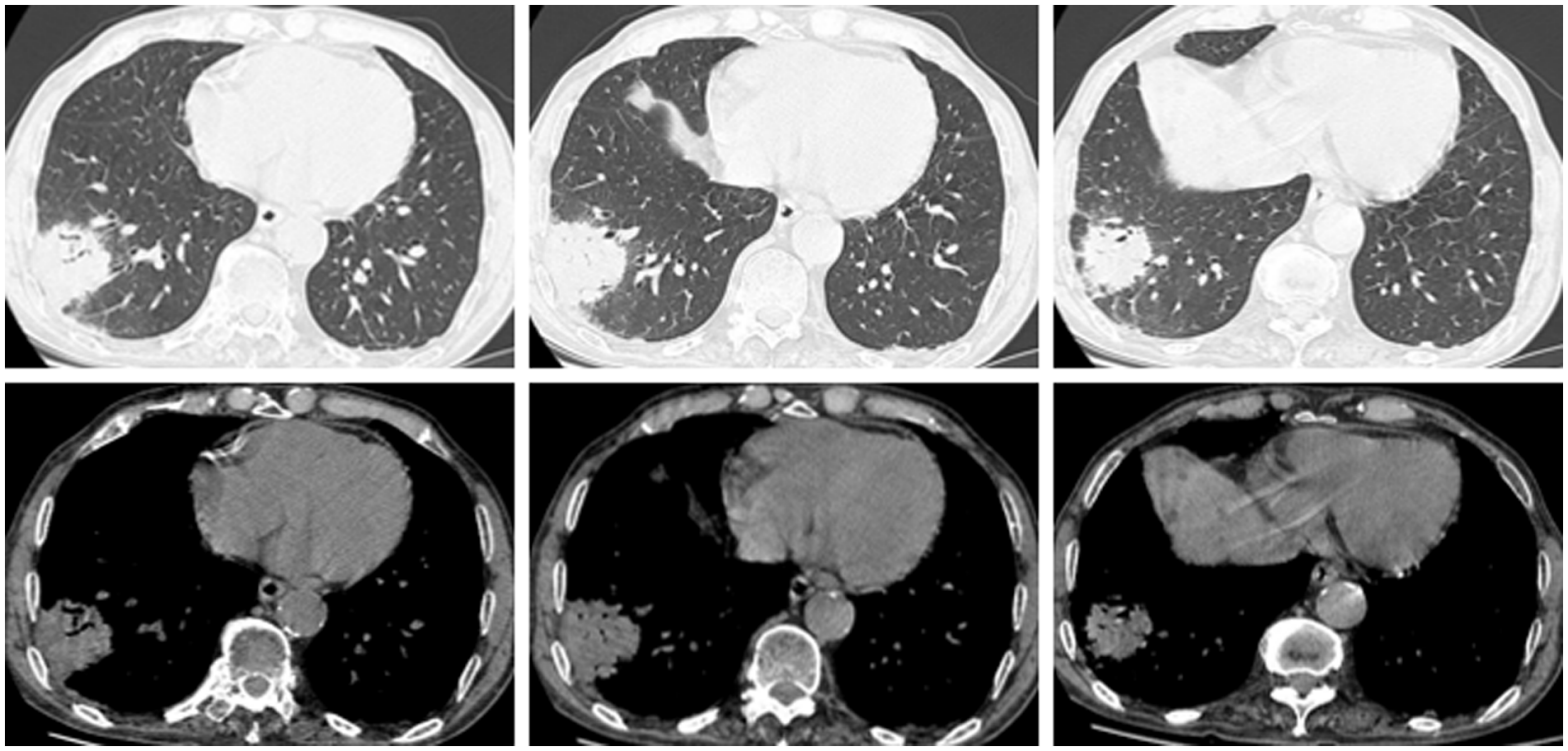
A patchy high-density shadow with blurred margins, approximately 4.5 mm in diameter, is observed in the lower lobe of the right lung. Air bronchogram sign is visible.

An 80-year-old male patient with end-stage renal disease due to polycystic kidney disease had undergone maintenance hemodialysis for 6 months. He was admitted with a 2-day history of fever, cough, and sputum production. Past medical history included hypertension, coronary heart disease, and chronic bronchitis. Admission vital signs: Temperature 39.2 °C, blood pressure 117/59 mmHg. The patient appeared coarse breath sounds bilaterally but no rales or pleural friction rub. The left upper limb arteriovenous fistula had a palpable thrill and loud bruit. No lower limb edema. Admission laboratory findings: White blood cell count 6.30 × 10^9^/L, neutrophils 89% (↑), hemoglobin 106 g/L (↓), platelets 154 × 10^9^/L; C-reactive protein 176.24 mg/L (↑), procalcitonin 2.8 ng/mL (↑); erythrocyte sedimentation rate 24 mm/h; serum sodium 131.1 mmol/L (↓), chloride 98.3 mmol/L (↓), blood urea nitrogen 14 mmol/L, creatinine 477.9 μmol/L (↑), albumin 32.6 g/L (↓); D-dimer 659 μg/L (↑). Chest CT at admission revealed an infectious lesion in the right lower lobe (Figure 1).

**Figure 2:**
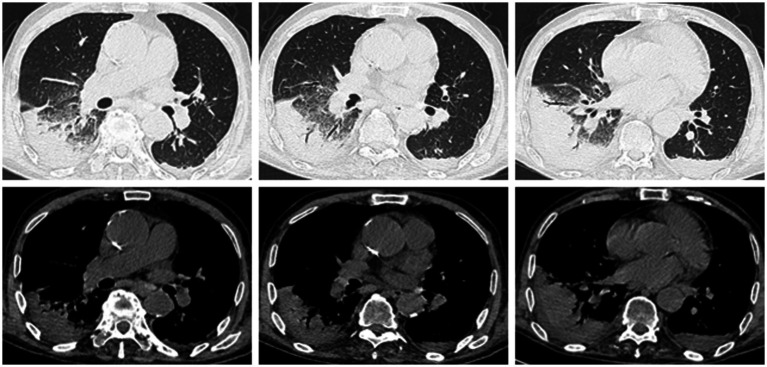
The middle lobe of the right lung, the lower lingular segment of the left upper lobe, and the bilateral dorsal weight-bearing surfaces show patchy consolidation with linear streaks, within which air-filled bronchial shadows are visible. Patchy ground-glass opacities surround the right lung lesion. The anterior segment of the right upper lobe exhibits patchy consolidation with surrounding ground-glass opacities, exhibiting irregular morphology and blurred margins.

Following admission, empirical antimicrobial therapy (cefoperazone/sulbactam) was started on day 1 for suspected community-acquired pneumonia, aiming to cover common respiratory pathogens such as *Streptococcus pneumoniae* and *Haemophilus influenzae*. Blood cultures, sputum cultures, and relevant virological tests were collected. On day 3, fever persisted at 39.2 °C and CRP rose to 260.6 mg/L with neutrophils at 92.6%. Respiratory consultation raised concerns for fungal infection, tuberculosis, or malignancy. Treatment was escalated to imipenem/cilastatin 1.0 g every 12 h combined with vancomycin 0.5 g every 12 hsupplemented with voriconazole 0.2 g every 12 h for fungal coverage. Persistent high fever continued, routine microbiological investigations (blood cultures, sputum cultures, PCR for common respiratory viruses and atypical pathogens, TORCH, G/GM tests) were all negative and on the night of the third day, the patient developed significant neuropsychiatric abnormalities, presenting with persecutory delusions and agitation. Protective restraints were applied. A lumbar puncture was not performed given the patient’s extreme agitation, anticoagulation requirements during CRRT, and absent meningeal signs. On the fourth day, the patient developed Type I respiratory failure and was transferred to the nephrology intensive care unit for continuous renal replacement therapy (CRRT). A follow-up chest CT on the fifth day after admission showed multiple inflammatory lesions in the right lung and left lower lobe; pleural effusion, which had progressed since the previous scan (Figure 2). Faced with this diagnostic impasse,the attending physician conducted a thorough history review and the patient told him that he had kept parrots for 1 month prior to admission. This critical clue immediately suggested a potential zoonotic disease. Metagenomic next-generation sequencing (mNGS) was promptly performed on blood and sputum samples. Results revealed abundant *Chlamydia psittaci* sequences in both blood and sputum samples (sputum additionally detected *Lactobacillus rhamnosus*, which was assessed as likely commensals/colonizers).

**Figure 3:**
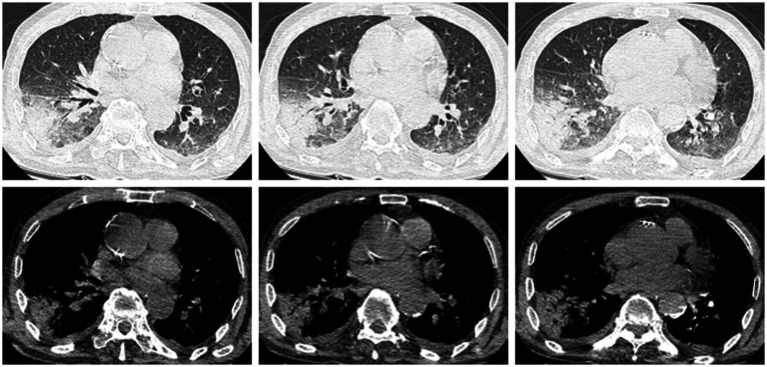
A patchy consolidation shadow is visible in the lower lobe of the right lung, with some areas showing signs of air-filled bronchi.

**Figure 4:**
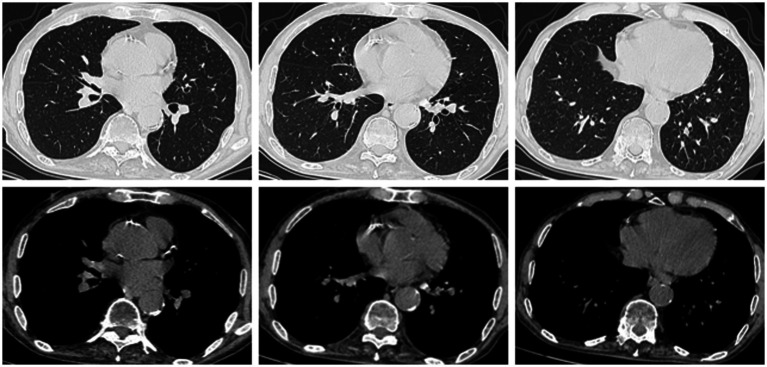
A small patch of faint opacity in the lower lobe of the right lung.

Following confirmation of the diagnosis, targeted antimicrobial therapy was promptly initiated: oral minocycline 100 mg twice daily combined with intravenous azithromycin 0.5 g daily. After treatment adjustment, the patient’s temperature normalized within 4 days, inflammatory markers (CRP, PCT) rapidly decreased, and neuropsychiatric symptoms gradually resolved. After approximately 10 days of treatment, the patient’s condition showed significant improvement. A follow-up chest CT scan revealed that the infectious lesion in the right lung had decreased in size compared to the previous scan (Figure 3). The patient was discharged successfully on day 16 on oral minocycline 100 mg twice daily plus cefdinir. A follow-up chest CT scan 10 days after discharge showed marked improvement in the original infectious lesion (Figure 4).

## Discussion

For refractory infections in immunocompromised hosts, proactive screening for uncommon and zoonotic pathogens is essential: Patients undergoing maintenance hemodialysis represent a classic immunocompromised population, with infection being a leading cause of mortality. When confronting severe pneumonia unresponsive to conventional antimicrobial therapy and with an unknown pathogen, clinical reasoning must extend beyond common pathogens. The core lesson from this case is: Detailed, repeated epidemiological history taking (especially regarding exposure to pets or birds) is the first key to unlocking diagnostic dilemmas. This simple yet critical step is often overlooked in busy clinical practice. An Italian study involving 76 patients found that most cases of psittacosis were associated with a history of contact with poultry (1).

Timely application of mNGS technology provides rapid pathogen identification for complex critical infections: *Chlamydia psittaci* is difficult to isolate via traditional culture, and serological testing may exhibit delays and false negatives (2). In this case, after conventional tests were negative and empirical treatment failed, prompt submission for mNGS enabled rapid, unbiased pathogen identification. For immunocompromised patients, severe, complex, or emerging rare infections, mNGS serves as a preferred first- or second-line diagnostic tool, demonstrating significant value in guiding targeted therapy and improving outcomes. Multiple recent case series have further demonstrated the sensitivity and speed of mNGS in diagnosing *Chlamydia psittaci* pneumonia, particularly when conventional methods fail (3–6).

However, mNGS results require careful interpretation. Clinicians must distinguish true pathogens from contamination or colonization by integrating: (A) specimen type (sterile vs. non-sterile sites); (B) consistency across multiple specimen types; (C) clinical compatibility with the patient’s presentation; (D) epidemiological plausibility; and (E) response to targeted therapy. Unfortunately, direct testing of the parrots was not feasible in our case, as the birds had been removed from the household by the time the diagnosis was established and the family was unable to provide samples for testing. We have acknowledged this as a limitation. However, the detection of *Chlamydia psittaci* in both blood and sputum, together with clinical and epidemiological congruency and rapid therapeutic response, confirmed it as the true pathogen, while *Lactobacillus rhamnosus* in sputum were considered likely colonizers.

Psittacosis manifests beyond “atypical pneumonia”. Psittacosis can cause severe central nervous system involvement, such as delirium and psychotic symptoms, which may even present as prominent or initial manifestations (7). A study from the United Kingdom reported that 2 out of 156 patients developed encephalitis (8). Cerebellar dysfunction may be prominent. Psychiatric symptoms and increased intracranial pressure have been reported (9). In rare cases, true meningitis with cerebrospinal fluid leukocytosis may occur. Positive results from cerebrospinal fluid metagenomic sequencing have been reported (10). One patient who developed status epilepticus experienced prolonged severe neurological disease. The presence of Chlamydia DNA in cerebrospinal fluid confirmed direct invasion of the central nervous system by Chlamydia (11).

The pathogenesis is thought to involve both direct invasion of the central nervous system by the organism and immune-mediated mechanisms. Risk factors for severe or atypical presentations include advanced age, immunocompromised status, delayed antimicrobial therapy, and high bacterial load with systemic dissemination. In our patient, the neuropsychiatric symptoms were prominent and preceded the onset of respiratory failure, serving as an early indicator of severe disease. Clinicians should recognize that psittacosis should be included in the differential diagnosis for critically ill pneumonia patients with unexplained neuropsychiatric symptoms.

## Data Availability

The original contributions presented in the study are included in the article/supplementary material, further inquiries can be directed to the corresponding author.
